# Infection of the Conceptus With African Swine Fever Virus Following Artificial Insemination in Sows: A Pathological Study

**DOI:** 10.1155/tbed/7164197

**Published:** 2026-06-29

**Authors:** Julia Sehl-Ewert, Virginia Friedrichs, Sandra Blome

**Affiliations:** ^1^ Department of Experimental Animal Facilities and Biorisk Management, Friedrich-Loeffler-Institut, Südufer 10, Greifswald-Insel Riems, 17493, Germany, fli.de; ^2^ Institute of Diagnostic Virology, Friedrich-Loeffler-Institut, Südufer 10, Greifswald-Insel Riems, 17493, Germany, fli.de

**Keywords:** African swine fever, artificial insemination, conceptus, pathology, placenta, sow, vertical transmission, virus

## Abstract

African swine fever virus (ASFV) has recently been shown to be transmissible via artificial insemination (AI) with contaminated semen. In this study, we performed a comprehensive pathological investigation to assess vertical transmission and infection of the conceptus. A total of 14 gilts were experimentally inseminated and examined by clinical monitoring, virological analyses, gross and histopathology, immunohistochemistry, and combined RNAScope in situ hybridization and immunofluorescence to localize viral antigen and nucleic acids and to characterize infected cell populations. ASFV infection of conceptuses was demonstrated, supporting the possibility of vertical transmission. Viral antigen and nucleic acids were widely distributed across multiple tissues of the conceptus, indicating systemic infection. Infection patterns showed considerable heterogeneity, with variable viral load, morphology, and antigen detection observed both between animals and among conceptuses from the same uterus. Gross examination revealed uterine alterations, including hyperemia, edema, and accumulation of intrauterine fluid, frequently associated with embryonic loss or remnants of extraembryonic membranes. In the female reproductive tract, viral antigen was detected in multiple compartments, including the ovary, uterus, cervix, and vagina. Notably, viral antigen was detected in either maternal or fetal compartments but not simultaneously, indicating a temporally dynamic and spatially distinct pattern of infection and suggesting that multiple routes of maternal‐to‐conceptus transmission may be involved. Colocalization studies in fetal tissues revealed that ASFV‐infected cells included subsets expressing macrophage‐associated markers such as CD163 and Iba‐1, while a substantial proportion of infected cells could not be phenotypically assigned, indicating a broader and incompletely defined cellular tropism. Together, these findings provide new insights into ASFV transmission biology and pathogenesis in early gestation and highlight the complexity of maternal‐to‐conceptus transmission, warranting further investigation of infection dynamics at the feto‐maternal interface and the identity of fetal target cell populations.

## 1. Introduction

African swine fever (ASF) is a systemic hemorrhagic and notifiable animal disease that has become a serious global problem in the domestic pig industry and wild boar population [1]. The causative agent, African swine fever virus (ASFV), is the sole representative of the family *Asfarviridae*. Outside the sylvatic cycle in Africa, ASFV is transmitted through direct and indirect contact. Contaminated blood as well as infectious secretions and excretions such as saliva, urine, and feces represent important sources of horizontal transmission. Transmission occurs predominantly via the oronasal route, whereas aerosol transmission is considered of minor importance. In addition to these well‐established routes, the role of reproductive pathways in ASFV transmission has only recently gained attention [2–4].

Artificial insemination (AI) is widely used in modern pig production and represents a highly efficient tool for genetic dissemination. However, it may also facilitate the spread of infectious agents over long distances if contaminated semen is used [5]. Recently, Friedrichs et al. [2] demonstrated that ASFV can be transmitted via AI with infected semen.

In this experimental study, adult boars were intramuscularly (i.m.) inoculated with the moderately virulent ASFV strain “Estonia 2014.” Insemination with ASFV‐positive semen resulted in a direct infection in a large proportion of gilts, while the remaining animals were most likely infected through contact. All animals subsequently developed clinical diseases. Pregnancy was confirmed in the majority of animals. Notably, viral genome and infectious virus were detected not only in the inseminated gilts but also in their conceptuses, including aborted embryos and implanted fetuses, suggesting efficient transmission from the sow to the conceptus.

Evidence for vertical or transplacental transmission of ASFV has previously been reported under both field conditions [6] and experimental settings [7, 8]. However, these studies primarily relied on virological detection methods and reported only limited detection of viral genomes in fetal tissues. As a result, the pathological features of fetal infection, the distribution of viral antigen within the maternal reproductive tract and conceptuses, and the identity of infected cell populations remain poorly understood. In particular, it is unclear which cell types support viral replication at the early stages of gestation and how maternal infection relates to fetal involvement.

In the present follow‐up study, we therefore performed a comprehensive pathological investigation of ASFV infection in gilts following AI with contaminated semen. Particular emphasis was placed on the female reproductive tract and conceptuses, including the assessment of gross and histomorphological lesions, localization of viral antigens and nucleic acids, and characterization of infected cell populations. This approach aimed to provide deeper insights into the mechanisms of vertical transmission and ASFV pathogenesis during early pregnancy.

## 2. Material and Methods

### 2.1. Study Design

The study was performed in accordance with the current German Animal Welfare regulations and approved by the competent authority (Landesamt für Landwirtschaft, Lebensmittelsicherheit und Fischerei Mecklenburg‐Vorpommern [LALLF M‐V]) under Reference Number 7221.3‐1‐071/21.

The recently published study was conducted to investigate the transmission of ASFV to sows via AI [2]. The samples from this study were subjected to detailed pathomorphological analysis to trace the transmission of the virus from the sow to offspring.

### 2.2. Animal Experiment

Details of the experimental design can be read in the recently published study about the transmission of ASFV from infected boars to gilts after AI [2].

Briefly, in total, four mature boars (*Sus scrofa domesticus*) were successfully inoculated i.m. with a spleen suspension containing 1 × 10^4^ hemadsorbing units 50% (HAU_50_) of the ASFV strain “Estonia 2014” (gt II). Semen from infected boars was obtained at days 4 and 5 post i.m. inoculation was used for standard AI of 14 gilts, ear‐tagged with #530, #533, #534, #536, #539, #567, #576, #610, #614, #627, #630, #635, #646, and #654. The 14 gilts were housed in two separate pens (pen 1 and pen 2) for management, animal welfare, and biosecurity reasons. Both pens were clustered within the same stable unit in the high‐containment facility. Allocation to pens was done prior to insemination, and all animals were subjected to the same infection protocol. Thus, pens did not represent distinct experimental treatment groups but housing units with separate contact networks. Ten out of 14 gilts were inseminated twice, whereas others (#536, #610, #627, and #630) were only inseminated once. AI was performed with pooled semen obtained from all four ASFV‐infected boars, as described previously [2]. Because successful AI depends on gilt cooperation and adequate estrus behavior, not all animals could be inseminated twice as intended, which may introduce some variability in exposure but reflects typical conditions in practice and was considered in the interpretation of infection and fetal outcomes. During the trial, all animals were daily scored according to the protocol by Mittelholzer et al. [9] with slight modifications. Animals were humanely euthanized when they reached the humane endpoint (clinical score of 15) or in case of other intolerable conditions [10]. The end of the experiment was set at 35 days postinsemination, with two sows being removed from the experiment 9 days postinsemination as planned to investigate the early phase of pathological manifestation after infection. This time point was selected to assess initial pathological changes during the phase of high viremia and early systemic viral dissemination.

### 2.3. Detection of ASFV Genome and Infectious Particles

#### 2.3.1. Sample Collection and Processing

To assess ASFV genome loads and the presence of infectious virus, a broad range of tissues was collected from all gilts at necropsy, including blood, tonsil, mandibular lymph node (mnLN), lung, kidney, spleen, liver, gastrohepatic lymph node (ghLN), inguinal lymph node (ingLN), popliteal lymph node (pLN), and samples from the female reproductive tract (vestibulum, vagina, cervix, uterus, salpinx, and ovaries). In addition, all aborted conceptuses that could be retrieved were collected, as well as conceptuses from gilt #533, which remained pregnant until the end of the study (36 days postinsemination). Tissue samples were homogenized in sterile 1× PBS using a TissueLyzer II (Qiagen) with metal beads. Conceptuses were processed under sterile conditions by repeated washing in PBS, followed by homogenization of the tissues. Aborted conceptuses originated from gilts #627, #639, and #654.

#### 2.3.2. ASFV Genome Detection by qPCR

DNA was extracted from 100 µL of whole blood or tissue homogenate using the NucleoMag VET Kit (Macherey‐Nagel) on a KingFisher 96 Flex System (Thermo Fisher Scientific) according to the manufacturer’s instructions. An internal control provided in the qPCR kit was included in all extraction reactions. Quantitative PCR was performed using the virotype ASFV PCR kit (Indical) on a CFX96 Real‐Time PCR System (Bio‐Rad). Because viral genome loads in conceptus tissues were expected to be low, a qPCR assay with higher reported analytical sensitivity in this matrix was used for these samples, whereas a standard, widely used assay was applied to maternal blood and organ tissues [11]. Therefore, for conceptuses, the VetMax ASFV Detection Kit (ThermoFisher) was used. Data were analyzed using GraphPad Prism 9 (GraphPad Software Inc.).

#### 2.3.3. Virus Isolation

The presence of infectious ASFV particles in reproductive organs and conceptuses was assessed by the hemadsorption test (HAT) as previously described [2]. HATs were used to determine the presence of an infectious virus in tissue samples of gilts and conceptuses. Peripheral blood mononuclear cells were first seeded into 24‐well plates and differentiated into macrophages by adding recombinant colony‐stimulating factor 2 (CSF2) at a concentration of 2 ng/mL. Then, 100 µL of tissue homogenate was added in duplicate to the differentiated macrophages. After an incubation period of 72 h, the plates were stored at −80°C for at least 24 h to ensure complete freezing and cell lysis. The resulting supernatants were subsequently examined by HAT to determine the presence of infectious ASFV. Each blind passage sample (*n* = 2 technical replicates) was analyzed in quadruplicate (*n* = 4 wells) in the HAT. Results were categorized as negative (−, all wells were negative) or positive (+, at least one well was positive for rosettes).

### 2.4. Pathology

#### 2.4.1. Necropsy

All animals were submitted to necropsy (Table 1). Scoring of gross lesions was performed according to the protocol published by Galindo‐Cardiel et al. [12] with recently published modifications [13]. Pathological alterations were evaluated based on an ordinal scale from 0 to 3 (normal [0], mild [1], moderate [2], or severe [3]). Organ samples (lung, liver, spleen, ghLN, kidney, ovary, salpinx, uterine body, cervix, vagina, vestibulum, and, if available, conceptuses) were fixed in 10% neutral‐buffered formalin for at least 3 weeks.

**Table 1:** Overview of gilts included in the study and corresponding day of euthanasia and sampling post insemination (dpi).

Gilt ID	Day of euthanasia/sampling (dpi)
539	9
567	9
536	17
534	20
627	33
530	35
533	35
614	35
576	35
646	35
635	36
630	36
610	36
654	36

### 2.5. Histopathology

#### 2.5.1. Histomorphology

Formalin‐fixed tissue samples were routinely processed, embedded in paraffin, sectioned at 2–3 µm, and stained with hematoxylin and eosin (H&E). Sections were examined for histopathological alterations. In the absence of a dedicated negative control group, interpretation of lesions was restricted to those spatially associated with viral antigen detection in order to ensure specificity and avoid overinterpretation of nonspecific findings.

#### 2.5.2. Immunohistochemistry

Consecutive sections were subjected to immunohistochemistry for the detection of ASFV antigen. An in‐house‐generated rabbit polyclonal antibody directed against ASFV p72 was used. Immunohistochemical staining was performed using the avidin–biotin–peroxidase complex (ABC) method. Detailed staining procedures have been described previously [14].

#### 2.5.3. RNA In Situ Hybridization and Immunofluorescence

To characterize ASFV‐infected target cells, colocalization studies were performed using either combined RNAScope in situ hybridization and immunofluorescence or double immunofluorescence labeling on consecutive tissue sections. RNAScope was carried out using the RNAscope 2.5 HD Reagent Kit‐Red (Advanced Cell Diagnostics, Newark, CA, USA) according to the manufacturer’s instructions. A custom probe targeting ASFV p72 nucleic acids (p72 mRNA and genomic DNA) was used. Positive (PPIB) and negative (DapB) control probes were included to verify the assay performance and specificity.

For combined RNAScope and immunofluorescence, sections were subjected to immunofluorescence staining following completion of the hybridization protocol. In addition, double immunofluorescence staining was performed to assess the colocalization of the ASFV p72 protein with selected cellular markers.

Following RNAScope treatment, sections were washed in PBS and permeabilized using 0.25% Triton X‐100 and 0.2% gelatin in PBS for 10 min at room temperature (RT). Sections were then blocked with 5% bovine serum albumin (BSA; Merck, A7979) for 60 min at RT and subsequently incubated with primary antibodies (Table 2) diluted in 1% BSA overnight at 4°C. After washing, sections were incubated with fluorophore‐conjugated secondary antibodies diluted in 1% BSA for 1 h at RT in the dark. Depending on the primary antibody, goat anti‐rabbit Alexa Fluor 488 (Invitrogen; 1:1000) or donkey anti‐rat Alexa Fluor 488 (Jackson ImmunoResearch; 1:200) was used. Nuclei were counterstained with Hoechst (1:20,000 in PBS, 5 min, in the dark). To reduce background fluorescence, sections were treated with a light‐protected solution of 10 mM copper(II) sulfate and 50 mM ammonium chloride, rinsed with deionized water, and mounted using Immu‐Mount (Fisher Scientific GmbH, Schwerte, Germany). All sections were examined by fluorescence microscopy.

**Table 2:** Primary antibodies used for immunohistochemistry and immunofluorescence.

Marker	Antibody, catalog number	Species	Mono‐/polyclonal	Dilution	Antigen retrieval	Supplier
ASFV p72	ASFs GST p72A	Rabbit	Poly	1:1600	Tris‐EDTA, pH = 9, 20 min, HIER	In‐house
Macrophage/monocyte	CD68, #ab955	Mouse	Mono	1:50	Citrate, pH = 6, 20 min, HIER	Abcam, Cambridge, UK
	CD163, #ab87099	Rabbit	Poly	1:50	Tris‐EDTA, pH = 9, 20 min, HIER	Abcam, Cambridge, UK
	MAC387, #E026	Mouse	Mono	Ready to use	Pronase, 1 h, RT	Linaris, Dossenheim, GER
	Iba‐1, #019‐19741	Rabbit	Poly	1:500	Citrate, pH = 6, 20 min, HIER	FUJIFILM Cellular Dynamics, Madison, WI, USA
Granulosa cell	Inhibin‐α, # bs‐1032R	Rabbit	Poly	1:100	Proteinase K, 30 min, 37°C	Bioss Inc., Woburn, MA, USA
Stem cell	SOX2, #14‐9811‐82	Rat	Mono	1:100	Citrate, pH = 6, 20 min, HIER	Thermo Fisher Scientific, Waltham, MA, USA
Mesenchymal cell	Vimentin, clone 9 #M0725	Mouse	Mono	1:40	Citrate, pH = 6, 20 min, HIER	Agilent, Santa Clara, CA, USA

*Note:* Antibodies were applied as indicated, including host species, clonality, dilution, antigen retrieval, and supplier. Heat‐induced epitope retrieval (HIER) was performed using citrate buffer (pH 6) or Tris/EDTA buffer (pH 9) for 20 min unless otherwise stated. Enzymatic antigen retrieval was carried out using pronase at room temperature (RT) or proteinase K at 37°C, as specified. Ready‐to‐use antibodies were applied without further dilution. Antibodies were selected based on prior use in porcine tissues or cross‐reactivity data provided by the manufacturers. The ASFV p72 antibody (ASFs GST p72A) was generated in‐house. Immunohistochemical staining was performed using the avidin–biotin–peroxidase complex (ABC) method.

## 3. Results

### 3.1. Clinical Disease in Gilts

Clinical disease progression has been described in detail previously [2]. Briefly, following IAI with ASFV‐contaminated semen, gilts developed typical signs of ASFV infection, including fever, lethargy, reduced feed intake, and skin discoloration. Differences in infection dynamics were observed between pens, with a delayed onset of clinical signs in animals in pen 2 (Figure 1). The blood of all gilts was tested in qPCR at 7 days post AI, which revealed that all except gilt #533 were successfully infected by AI, whereas only gilt #610 was successfully infected in pen 2. All gilts that were negative by blood qPCR at 7 dpi, including gilt #533, eventually contracted the virus from infected pen mates and developed delayed infection kinetics compared with animals infected directly via AI. Gilt #536 had to be euthanized on day 17 pi due to acute pulmonary failure. Gilts #539 and #567 were euthanized on day 9 pi to allow pathological evaluation of the organs in the phase of high viraemia (clinical scores were 4 and 8.5, respectively). Gilt #627 started showing clinical signs 26 days after insemination and reached the humane endpoint at day 33 with a cumulative score of 14.5. Most gilts aborted or resorbed conceptuses following the onset of fever. In contrast, gilt #533 remained pregnant until the end of the study (36 days postinsemination), while gilt #530 showed no evidence of conceptus implantation.

**Figure 1:**
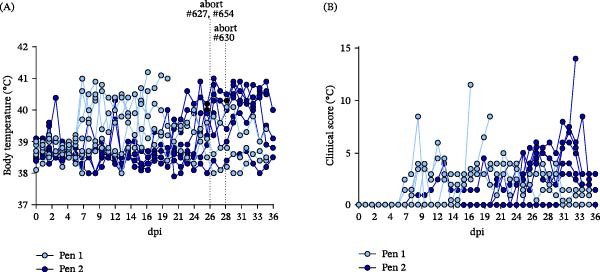
Rectal temperatures (A) and clinical scores (B) of gilts after artificial insemination with ASFV‐containing semen. All animals in pen 1 are depicted in light blue; all animals in pen 2 are in dark blue. Each individual/assessment day is represented by a symbol.

### 3.2. Virology

#### 3.2.1. Detection of ASFV Genome and Infectious Particles

Detailed virological results have been published previously [2]. In brief, the ASFV genome was detected in all examined organs of infected gilts (Figure 2), with no major differences between pens. In contrast, conceptus‐associated findings were heterogeneous. Aborted conceptuses showed variable viral genome loads, whereas conceptuses from gilt #533 exhibited marked differences within the same uterus: morphologically altered conceptuses were strongly positive by qPCR and virus isolation, while healthy‐appearing littermates were negative. An infectious virus was consistently detected in the female reproductive tract, including vagina, cervix, uterus, and salpinx, and in most ovarian samples (Table 3). Virus isolation from conceptuses corresponded with qPCR results, with only highly positive samples yielding an infectious virus.

**Figure 2:**
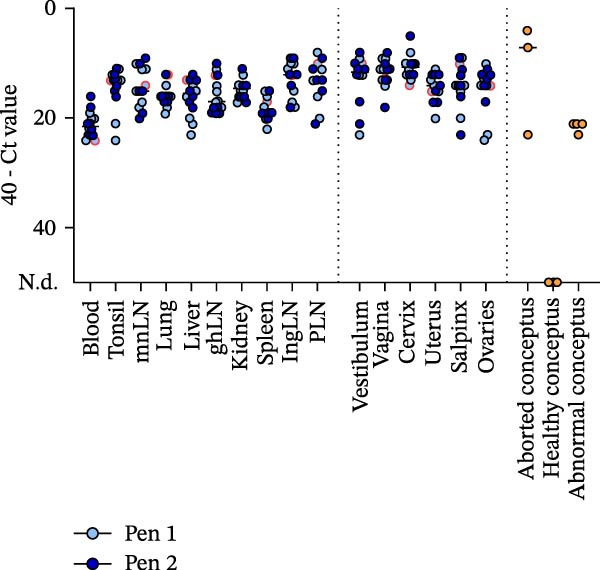
Assessment of ASFV genome loads in organs of gilts and aborted/retrieved conceptuses. Results are presented as 40‐Ct values, with higher values indicating higher ASFV genome loads. Each symbol represents an individual sample. Animals in pen 1 are shown in light blue, while animals in pen 2 are shown in dark blue. Conceptuses are indicated in orange. The graph includes the median. Gilt #533 is indicated by red‐bordered points.

**Table 3:** Results of virus isolation (HAT) of female reproductive organs and conceptuses.

Matrix	Gilts #													
	530	533	534	536	539	567	576	610	614	627	630	635	646	654
Vagina	+	+	+	+	+	+	+	+	+	+	+	+	+	+
Cervix	+	+	+	+	+	+	+	+	+	+	+	+	+	+
Uterus	+	+	+	+	+	+	+	+	+	+	+	+	+	+
Salpinx	+	+	+	+	+	+	+	+	+	+	+	+	+	+
Ovaries	+	+	+	−	−	+	+	+	+	−	+	+	+	+
Conceptus abort (*n* = 3)	—	—	—	—	—	—	—	—	—	−	+	—	—	−
Conceptus healthy (*n* = 3)	—	−	—	—	—	—	—	—	—	—	—	—	—	—
Conceptus abnorm (*n* = 4)	—	+	—	—	—	—	—	—	—	—	—	—	—	—

*Note*: −, negative result in HAT; +, positive result in HAT.

## 4. Pathology

### 4.1. Macroscopic Evaluation

#### 4.1.1. General Gross Lesions

As a result of AI with ASFV‐positive semen, all gilts showed pathological changes characteristic of ASF, regardless of the time of euthanasia and the severity of clinical signs. These changes mainly included ascites of varying degrees, pinpoint renal hemorrhages, hemorrhages and enlargement of lymph nodes, and pulmonary consolidation. Lesions, except for those of the reproductive organs, are shown in Figure 3. In addition, in 5 of the gilts (#627, #614, #635, #630, and #654) multiple renal infarctions were observed, and in one animal (#534) severe jaundice was diagnosed. Both findings were not included in the gross lesion score.

**Figure 3:**
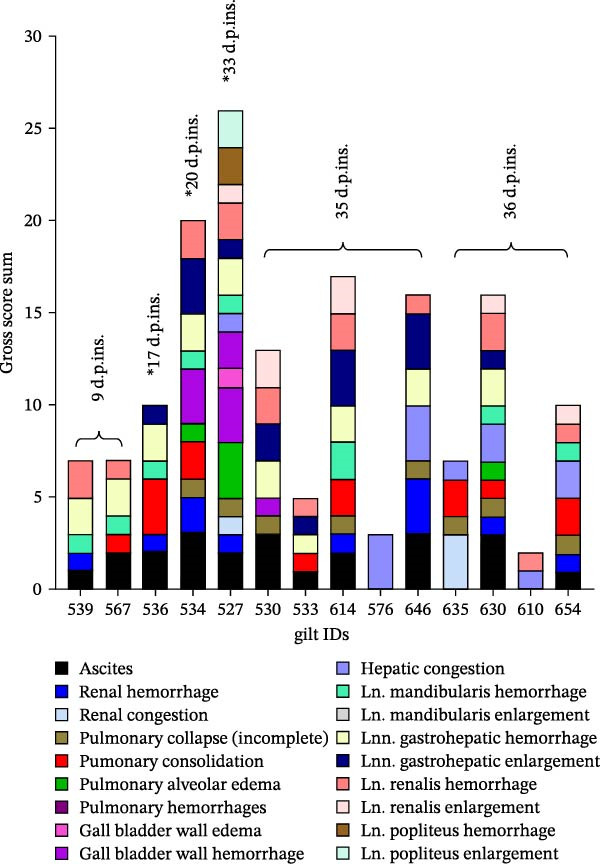
Presentation of the overall gross lesion score per animal. The day of euthanasia is indicated above the bars. Asterisks indicate gilts that reached the humane endpoint.

#### 4.1.2. Gross Lesions of the Female Reproductive Organs

Independent of the day of euthanasia, corpora lutea were present in all gilts except for animal #530. The ovaries and fallopian tubes were consistently free of lesions. On the gross examination of the uterus, particular attention was given to the presence of conceptuses and associated membranes (Table 4). Conceptuses or remnants of extraembryonic membranes were identified in animals #536, #534, #533, #614, and #654, whereas an aborted conceptus from animal #627 was recovered in the stable. In animals #536, #534, and #533, intact conceptuses as well as gray‐brownish membranous remnants were observed, while in #614 and #654, only residual fetal membranes were present. The majority of gilts showed uterine alterations (Figure 4A–F), including hyperemia and hemorrhages of the endometrium, as well as edematous uterine walls (Figure 4A,C). In animals euthanized due to severe clinical disease (#536, #534, and #627), the uterine lumen contained large amounts of fluid. In particular, animal #534 showed a purulent exudate admixed with remnants of extraembryonic membranes (Figure 4B). In addition, the uterine lumen frequently contained remnants of extraembryonic membranes at different stages of degeneration (Figure 4D,E) as well as intact extraembryonic membranes enclosing dead conceptuses (Figure 4F). Vaginal alterations, including hyperemia and hemorrhages, were observed in two animals (#567 and #646). Detailed findings are summarized in Table 3.

**Figure 4:**
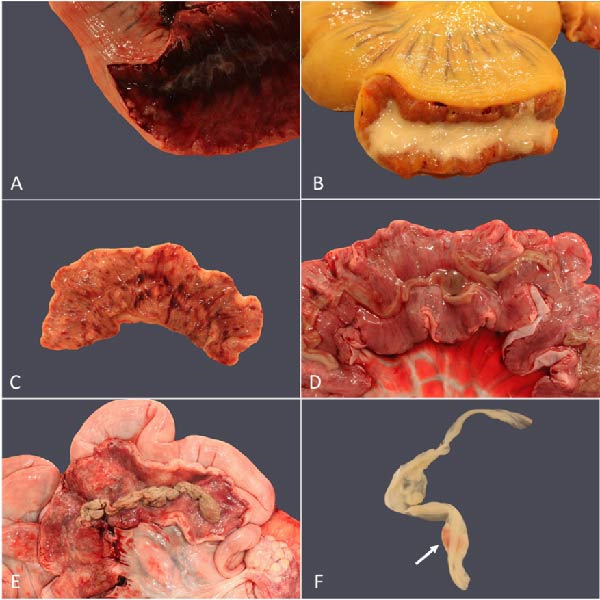
Gross uterine pathology of gilts following artificial insemination with ASFV‐infected semen. (A) Hyperemic and edematous uterine wall. The uterine lumen is distended and contains mucoid fluid admixed with thread‐like remnants of extraembryonic membranes. (B) Uterus of an icteric gilt filled with purulent exudate containing residues of embryonic membranes. (C) Endometrial mucosa with multifocal hemorrhages. (D, E) Uterine lumen containing extraembryonic membrane remnants of varying stages of degeneration. (F) Retrieved extraembryonic membrane enclosing a dead conceptus (arrow).

**Table 4:** Summary of gross pathological findings and reproductive status of gilts following artificial insemination with ASFV‐contaminated semen.

Gilt #	Necropsy (d.p.ins.)	Corpora lutea	Conceptus	Extraembryonic/fetal membranes	Uterine pathology	Vaginal/vulvar pathology
539	9	Present	Absent	Absent	None	None
567	9	Present	Absent	Absent	None	Multifocal pinpoint vulvar hemorrhages
536	17^a^	Present	Present^b^	Present	Uterus filled with dark red mucoid fluid	None
534	20^a^	Present	Present^b^	Present	Uterus filled with beige, creamy fluid; uterine wall edematous	None
627	33^a^	Present	Abortion confirmed (conceptus sampled in stable for histopathology)	Not applicable	Uterus filled with yellow serous fluid; endometrium diffusely hyperemic; mesometrium with oligofocal hemorrhages	None
530	35	Absent	Absent	Absent	None	None
533	35	Present	Present	Present	None	None
614	35	Present	Absent	Present	Endometrium diffusely hyperemic; uterine wall edematous	None
576	35	Present	Absent	Absent	None	None
646	35	Present	Absent	Absent	Endometrium diffusely hyperemic; uterine wall edematous	Vaginal mucosa diffusely hyperemic
635	36	Present	Absent	Absent	Endometrium diffusely hyperemic	None
630	36	Present	Abortion confirmed/no conceptus available	Absent	Endometrium diffusely hyperemic	None
610	36	Present	Absent	Absent	Endometrium diffusely hyperemic	None
654	36	Present	Abortion confirmed/no conceptus available	Present	Endometrium diffusely hyperemic	None

Abbreviation: d.p.ins., days post insemination.

^a^Euthanized due to severe clinical signs.

^b^Histological detection only.

#### 4.1.3. Gross Lesions of the Conceptuses

Macroscopic evaluation of conceptuses was only feasible in animal #533 (Figure 5). The fetal crown–rump length ranged from 1.0 to 3.8 cm.

**Figure 5:**
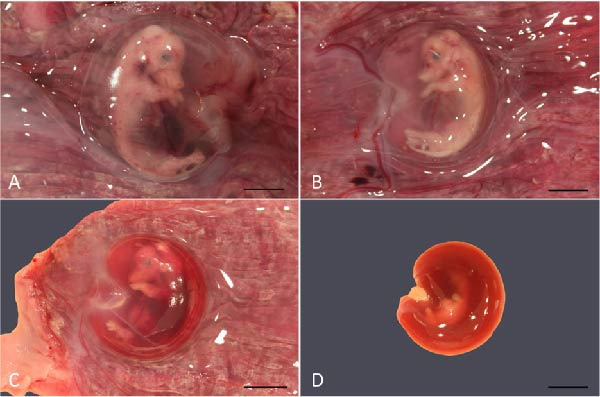
Macroscopic appearance of conceptuses retrieved from the same uterus. (A, B) Conceptuses without obvious macroscopic abnormalities. (C, D) Growth‐restricted conceptuses with altered amniotic sacs. Scale bars = 1.0 cm.

Growth‐restricted conceptuses (<3.6 cm) exhibited petechiae, ecchymoses, or diffuse reddening of the amniotic sac, whereas larger, normally developed conceptuses showed no macroscopic abnormalities (Figure 5A–D).

## 5. Histopathology

### 5.1. Immunohistochemistry

#### 5.1.1. Antigen Detection in Nonreproductive Organs and Female Reproductive Tract

Antigen was detected in animals #539, #567, #536, #627, and #530, while all other individuals were negative (Figure 6). Viral antigens were present in varying amounts. While abundant antigen‐positive cells were detected in the lung, spleen, and lymph nodes, the female reproductive tract was also affected.

**Figure 6:**
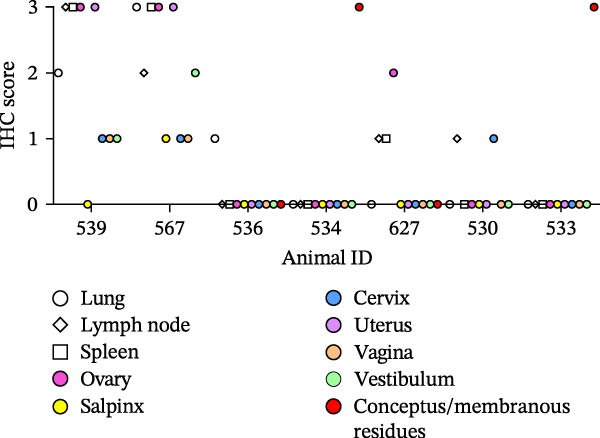
Semiquantitative scoring of the ASFV antigen‐positive cells in nonreproductive organ samples and the female reproductive tract as well as conceptuses and/or their membranous residues.

Representative immunohistochemical findings in the female reproductive organs are shown in Figure 7. High numbers of antigen‐positive cells were observed in the ovary and uterus, particularly in animals #539 and #567 examined at day 9 postinsemination (Figure 7B,C). In contrast, the cervix, vagina, and vestibulum generally exhibited lower numbers of antigen‐positive cells (Figure 7D–F).

**Figure 7:**
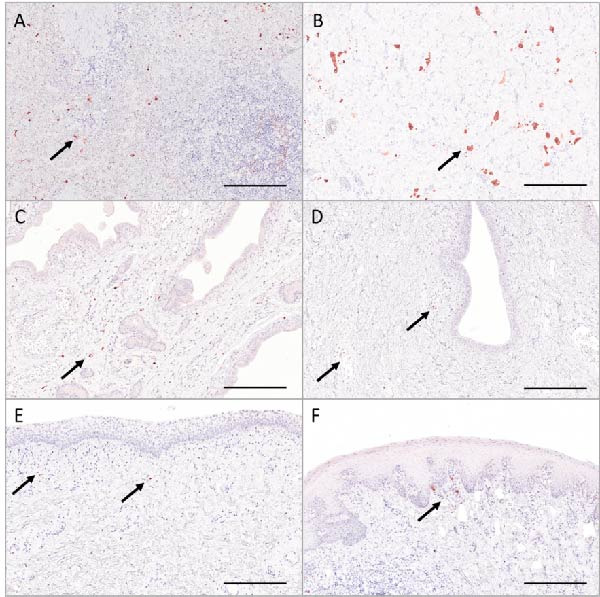
Representative immunohistochemical detection of the ASFV p72 antigen in gilts following artificial insemination with ASFV‐contaminated semen. (A) Spleen (reference organ) showing abundant antigen‐positive cells. (B) Ovary of a gilt at 9 days post insemination (d.p.ins.) with widespread antigen detection. (C) Uterus of a gilt at 9 d.p.ins. demonstrating numerous antigen‐positive cells. (D) Cervix, (E) vagina, and (F) vestibulum showing overall low numbers of antigen‐positive cells. Scale bars = 250 µm.

A more detailed overview of antigen localization within the female reproductive tract is provided in Table 5. Antigen‐positive cells were detected in the corpus luteum and ovarian stroma, in endothelial and vascular wall structures of the salpinx, and in the endometrial stroma, mucosal epithelium, and vascular wall of the uterus. In the cervix and vagina, the antigen was present in stromal and epithelial compartments, with additional detection in luminal cellular debris in the cervix. In the vestibulum, antigen was observed in stromal and epithelial compartments as well as in mucosa‐associated lymphoid tissue (MALT).

**Table 5:** Anatomical distribution of ASFV antigen within female reproductive organs.

Organ	Antigen localization
Ovary	Corpus luteum; ovarian stroma
Salpinx	Endothelium; vascular wall
Uterus	Endometrial stroma; mucosal epithelium, vascular wall
Cervix	Stromal tissue; mucosal epithelium; luminal cellular debris
Vagina	Stromal tissue; mucosal epithelium
Vestibulum	Stromal tissue; mucosal epithelium; mucosa‐associated lymphoid tissue (MALT)

In animals #536, #627, and #530, antigen was detected only in low amounts in individual organs, including the lung, spleen, or lymph node. In #627, positive cells were detected only in the ovary, whereas in #530, antigen was restricted to the cervix. All organs, including the reproductive tract, were negative in sows #534 and #533.

#### 5.1.2. Antigen Detection in Conceptuses

Although all examined maternal tissues were negative for ASFV antigen, conceptuses of #534 and several grossly altered conceptuses of #533 exhibited substantial amounts of viral antigen disseminated across tissues of the conceptus (Figure 8). Immunohistochemistry revealed widespread antigen‐positive cells throughout the conceptus, indicating systemic fetal infection (Figure 8A), with disseminated antigen‐positive cells present across multiple organ systems (Figure 8B). In contrast, the conceptuses of antigen‐positive animals #536 and #627 were negative.

**Figure 8:**
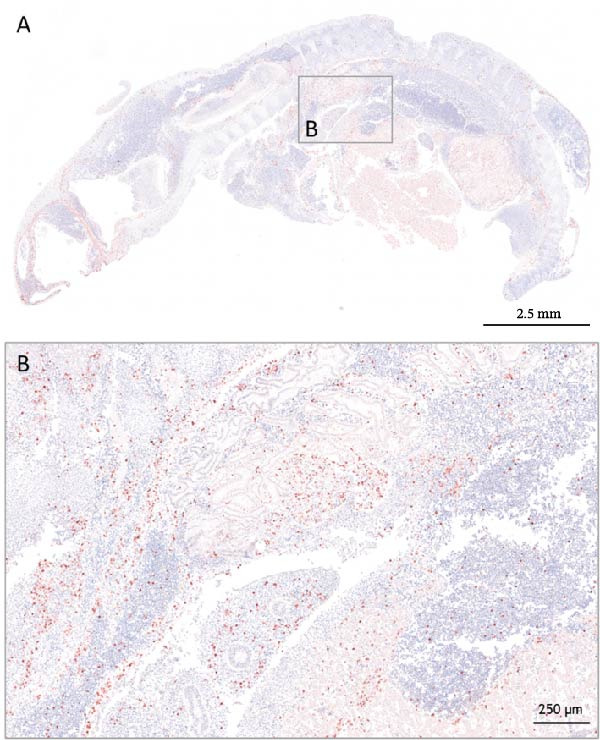
Immunohistochemical detection of the ASFV antigen in an infected conceptus. (A) Low‐power overview of a sagittal section demonstrating widespread antigen‐positive cells throughout the conceptus. (B) Higher magnification of the boxed area in panel (A) showing disseminated antigen‐positive cells across multiple fetal organs.

#### 5.1.3. Antigen Detection in the Placenta

Residues of extraembryonic/fetal membranes were detected in animal #534 (20 d.p.ins.) and #533 (35 d.p.ins.). In sow #533, detailed examination of the feto‐maternal interface revealed that viral antigen was exclusively present on the fetal side. Antigen‐positive cells were confined to the fetal placental mesenchyme, while no antigen was detected in the maternal compartment (Figure 9). In sow #534, antigen‐positive cells were likewise detected in the fetal compartment. The maternal side was present but detached and negative for viral antigens.

**Figure 9:**
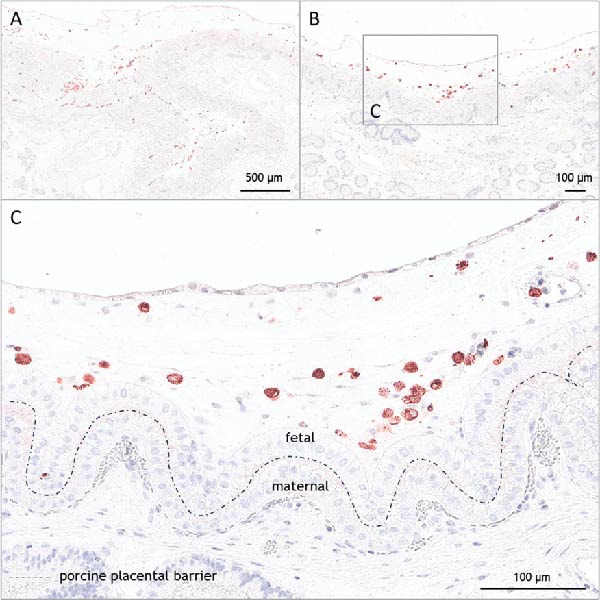
Immunohistochemical detection of the ASFV p72 antigen in the placenta of sow #533. (A) Low‐power overview demonstrating disseminated antigen‐positive cells within placental tissue. (B) Intermediate magnification showing the feto‐maternal interface. (C) High‐power magnification of the feto‐maternal interface. The dashed line indicates the porcine placental barrier separating fetal and maternal compartments. Antigen‐positive cells are confined to the fetal side, whereas the maternal side remains negative.

## 6. Immunofluorescence and RNAScope In Situ Hybridization

### 6.1. Identification of ASFV Infected Target Cells

Combined RNAScope in situ hybridization for the ASFV p72 nucleic acid signal and immunofluorescence or double immunofluorescence labeling were performed to further characterize infected cells in the female reproductive tract and conceptuses. The results of colocalization analyses of ASFV p72 nucleic acids or proteins with selected cellular markers are summarized in Table 6.

**Table 6:** Colocalization of ASFV p72 nucleic acids or protein with selected cellular markers in reproductive tissues and conceptuses.

	Iba‐1	Mac387	CD68	CD163	Inhibin α	SOX2	Vimentin
Ovary	✔	n.e.	n.e.	n.t.	✔ ^∗^	n.t.	n.t.
Uterus	✔	n.e.	n.e.	n.t.	n.t.	n.t.	n.t.
Conceptus	✔ ^∗^	n.e.	n.e.	✔	n.t.	n.e.	✔

*Note:* Importantly, colocalization does not imply that all ASFV‐positive cells expressed the respective marker. ✔, colocalization detected; ✔ ^∗^, colocalization detected in a subset of animals; –, no specific colocalization detected.

Abbreviations: n.e., not evaluable due to the absence of the respective target cell population in the examined tissue; n.t., not tested.

#### 6.1.1. Female Reproductive Tract

In the ovary and uterus, ASFV p72 colocalized with Iba‐1‐positive cells (Figure 10A,C). Colocalization with inhibin‐α‐expressing granulosa cells was observed in the ovary of #627 but not in #539 and #567 (Figure 10B). No specific colocalization was detected with MAC387 or CD68, while SOX2 and vimentin were not tested in these tissues. Importantly, not all ASFV‐positive cells colocalized with Iba‐1 or inhibin‐α, suggesting that ASFV infects a broader spectrum of cell types than those detected by the marker panel applied in this study.

**Figure 10:**
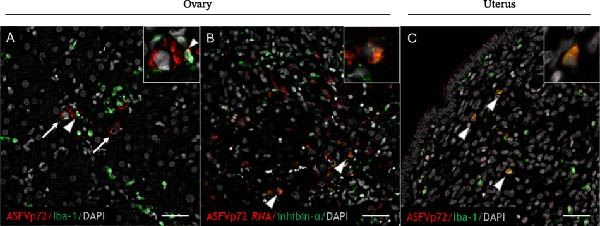
Colocalization of the ASFV p72 protein or nucleic acid signal with cellular markers in the ovary and uterus. Representative images of double immunofluorescence (panels A and C) and combined RNAScope in situ hybridization and immunofluorescence (panel B). The ASFV p72 protein or nucleic acid signal is shown in red, cellular marker proteins in green, and nuclei are counterstained with DAPI (gray). (A) Ovary showing colocalization of p72 protein with Iba‐1‐positive cells (arrowheads). The majority of larger ASFV‐infected cells lack Iba‐1 expression (arrow). (B) Ovary showing colocalization of p72 nucleic acid signal with inhibin‐α‐expressing granulosa cells (arrowheads). (C) Uterus showing colocalization of p72 nucleic acid signal with Iba‐1‐positive cells (arrowheads). Insets show higher magnification of selected areas, highlighting colocalization. Scale bars = 50 µm.

#### 6.1.2. Conceptuses

In conceptuses, p72 nucleic acids colocalized with CD163‐positive cells, while only limited colocalization was observed with Iba‐1 (Figure 11A,B). No specific colocalization was detected with MAC387, CD68, or SOX2. Minimal colocalization with vimentin was observed in infected fetal tissues (Figure 11C). Importantly, colocalization with CD163, Iba‐1, or vimentin was restricted to a subset of ASFV‐positive cells. A considerable proportion of p72‐positive cells did not express any of the tested markers.

**Figure 11:**
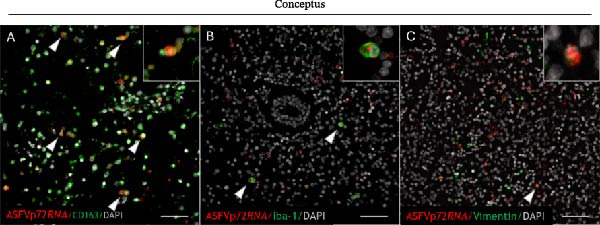
Colocalization of ASFV p72 nucleic acid signal with selected cellular markers in fetal tissue. Representative images of combined RNAScope in situ hybridization and immunofluorescence. The ASFV p72 nucleic acid signal is shown in red, cellular marker proteins in green, and nuclei are counterstained with DAPI (gray). (A) Colocalization of p72 nucleic acid signal with CD163. (B) Partial colocalization of p72 nucleic acid signal with Iba‐1. (C) Colocalization of p72 nucleic acid signal with vimentin. Arrowheads indicate representative double‐positive cells. Insets show higher magnification of selected areas, highlighting colocalization. Scale bars = 50 µm.

### 6.2. Histomorphological Investigation

#### 6.2.1. Female Reproductive Organs

The two sows, #539 and #567, which tested positive for the ASFV antigen throughout the female reproductive tract, were examined in more detail for histomorphological alterations. Ovaries and fallopian tubes were free of histopathological changes despite marked antigen detection in the ovaries. In the uterus, vascular congestion and mild multifocal lymphohistiocytic infiltrates were present in the subendometrial stroma, predominantly localized in a perivascular distribution and occasionally accompanied by perivascular single‐cell necrosis/apoptosis (Figure 12). These lesions were more pronounced in sow #539; however, in both animals, lesions were associated with viral antigens. In the vagina, lesions were markedly less pronounced and limited to few focal areas.

**Figure 12:**
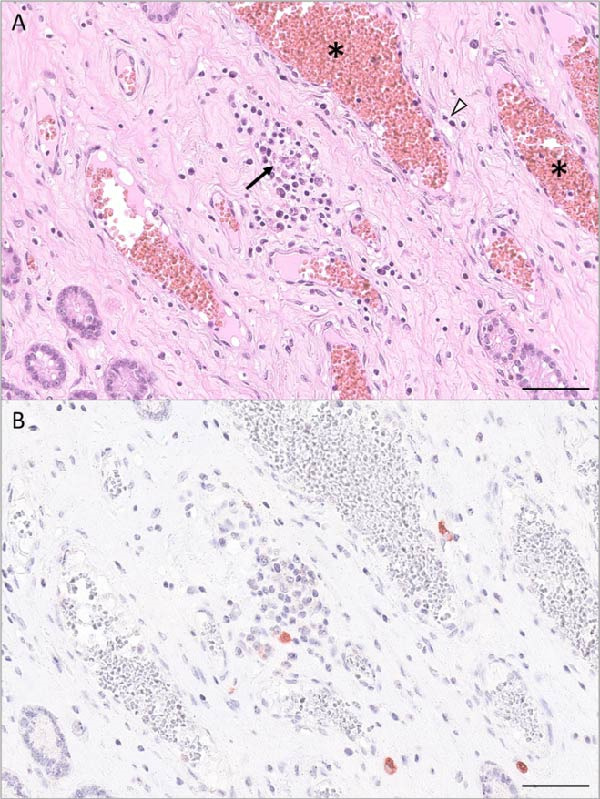
Histopathological and immunohistochemical findings in the uterus. (A) Subendometrial stroma showing vascular congestion ( ^∗^) and a perivascular lymphohistiocytic infiltrate with necrotic cellular debris (arrow). The arrowhead indicates single cells undergoing necrosis/apoptosis. (B) Consecutive section of panel (A) showing immunohistochemical staining for ASFV p72 antigen demonstrating scattered antigen‐positive cells, associated with the lesions. Scale bars = 50 µm.

#### 6.2.2. Conceptuses

Conceptuses from sow #534 were not subjected to histopathological evaluation due to advanced autolysis. In conceptuses from sow #533, autolytic changes were also present and precluded the reliable assessment of histopathological lesions.

To provide an integrated overview of clinical, virological, and pathological findings across all animals, key results are summarized in Supporting Information, Table S1.

## 7. Discussion

In this study, we provide pathological evidence, supported by virological data previously published by Friedrichs et al. [2], that ASFV can be transmitted from sow to conceptus in association with AI using ASFV‐contaminated semen. Using a combination of clinical monitoring, virological analyses, histopathology, viral nucleic acid, and antigen detection, we demonstrate that fetal infection was present even in the absence of detectable viral antigen in maternal tissues at the time of examination. Moreover, ASFV infection of conceptuses was highly heterogeneous, with variable morphology, viral genome loads, and antigen detection observed not only between animals but also among conceptuses originating from the same uterus. Due to the limited and heterogeneous sample size, as well as clustering of conceptuses within individual sows, these observations are presented descriptively and were not subjected to formal statistical analysis. Early evidence for the presence of ASFV in semen was provided by Thacker et al. [15], who demonstrated that semen collected from an infected boar was virus‐positive. These findings were subsequently confirmed and expanded by Roszyk et al. [3], who showed that semen derived from infected animals harbored infectious virus. Further studies reported extensive viral antigen distribution and pronounced histopathological alterations in the male reproductive organs, underscoring the reproductive tract as an important site of ASFV replication [4].

The present findings, together with previous data [2], demonstrate that AI with ASFV‐contaminated semen can result not only in systemic infection of gilts but also in disseminated infection of conceptuses. These observations were obtained using the moderately virulent ASFV strain Estonia 2014 [14, 16]. The prolonged clinical course associated with this strain enabled the establishment and progression of pregnancy and allowed the recovery of conceptuses and reproductive tissues for pathological investigation. In contrast, infection with highly virulent ASFV strains would likely result in rapid disease progression and early euthanasia, substantially limiting the opportunity to investigate maternal‐to‐conceptus transmission and conceptus pathology [17].

A striking observation was the presence of abundant viral antigen and viral nucleic acid signals in conceptuses despite the absence of detectable ASFV antigen in maternal tissues in selected animals. It should be noted that the applied RNAScope probe may detect both ASFV genomic DNA and viral mRNA. Consequently, the observed signal cannot be interpreted exclusively as evidence of active viral transcription. Importantly, animals euthanized at earlier time points exhibited clear ASFV antigen positivity within the reproductive tract, including the uterus and ovary. This apparent discrepancy likely reflects the temporal dynamics of ASFV infection rather than the true absence of maternal infection [18]. It is conceivable that maternal viral replication occurred transiently or locally within the reproductive tract at earlier time points and was subsequently cleared, whereas the virus persisted or replicated extensively within the tissues of the conceptus.

Notably, maternal ASFV antigen positivity was not invariably associated with conceptus infection as antigen‐positive gilts were identified in which all sampled conceptuses lacked detectable viral antigen. This is consistent with previous observations in pregnant minipigs infected with the Estonian ASFV strain, in which maternal infection did not result in detectable infection of fetuses during early to midgestation [16]. This contrasts observations made in PRRSV infection, where viral antigen is typically detected concurrently in both maternal and fetal compartments, particularly during late gestation [19].

The mechanisms underlying the dissociation between maternal and fetal viral antigen detection remain unclear. Several potential routes of maternal‐to‐fetal ASFV transmission are considered. These include hematogenous spread during transient maternal viremia, followed by transplacental transmission [20]. Alternatively, local viral replication within the female reproductive tract after AI may result in infection at the feto‐maternal interface [21]. In addition, infection of the very early conceptus prior to implantation cannot be excluded. Direct infection of the oocyte itself appears unlikely based on the existing literature [22]. This is supported by studies on other porcine viruses, such as PRRSV and porcine circovirus type 2 (PCV2), which have been detected in reproductive tissues but not in oocytes. In contrast, viral replication within embryonic cells after fertilization is possible and has been demonstrated for other porcine viruses, including PCV2 and classical swine fever virus [23, 24].

As described for other porcine viruses, fetal infection was highly heterogeneous in this study. In gilt #533, growth‐restricted and morphologically altered conceptuses were strongly positive for ASFV by qPCR, virus isolation, and antigen detection, whereas healthy‐appearing littermates from the same uterus were virus‐negative. Such intrauterine heterogeneity suggests that ASFV transmission to the conceptus is not uniform and may depend on local factors such as the implantation site, placental development, or microenvironmental differences at the feto‐maternal interface [25]. Comparable patterns have been reported for PRRSV, in which fetal outcomes are defined by resistance, resilience, or susceptibility despite uniform maternal exposure [26]. However, whereas PRRSV‐associated fetal death predominantly occurs during late gestation [20, 27], only early conceptuses were available in the present study. Factors such as target cell availability at the maternal‐fetal interface [28], localized virus‐induced tissue damage [29], and differences in placental development [26] may contribute to the heterogeneous infection patterns observed.

ASFV antigen and viral nucleic acid signals were widely distributed across all examined tissues in infected conceptuses, indicating systemic infection and rapid viral dissemination. Despite widespread infection, assessment of histopathological lesions was limited as autolytic changes were present or advanced in most tissues, precluding reliable evaluation of ASFV‐associated alterations in conceptuses.

Given that monocytes and macrophages are considered the primary target cells of ASFV in adult pigs [30], a panel of established myeloid and macrophage markers [31, 32] was employed to characterize infected fetal cells. Colocalization of ASFV p72 with CD163 and Iba‐1 was observed in a subset of infected fetal cells, whereas no specific staining was observed for the myeloid markers MAC387 or CD68. In addition, staining Sox2 did not yield detectable signals, and vimentin colocalization was observed only in a small subset of infected cells. Notably, the majority of ASFV‐positive cells did not colocalize with any of the tested markers, indicating that a substantial proportion of infected cells could not be phenotypically assigned. These findings contrast with PRRSV infection, where CD163^+^ macrophages represent the principal target cell population, and CD163 acts as a key receptor for viral entry [33, 34]. In the present study, however, CD163 expression was restricted to only a subset of infected cells, suggesting a broader or distinct cellular tropism of ASFV in early conceptuses. This may reflect the immature differentiation state of embryonic or fetal cells or the presence of yet uncharacterized target cell populations.

While the above findings relate to tissues of the conceptus, ASFV infection was also evident in multiple compartments of the female reproductive tract, including the vagina, cervix, uterus, salpinx, and ovaries. Previous evidence for ASFV presence in the female reproductive tract has been based on qPCR detection and virus isolation [2], whereas in the present study, viral antigen could be localized within defined anatomical structures. Detection of viral antigen at early time points postinsemination supports the concept that the female reproductive tract represents a site of local viral replication following exposure via semen.

In the ovary and uterus, ASFV p72 colocalized with Iba‐1‐positive cells, indicating infection of cells of the monocyte/macrophage lineage. However, a substantial proportion of ASFV‐positive cells, particularly larger cells within the corpora lutea, lacked Iba‐1 expression, suggesting that infection is not restricted to classical macrophage populations. Notably, colocalization with inhibin‐α‐expressing granulosa cells was observed in the ovary of one animal, indicating that ASFV may also infect nonmyeloid cell populations. Similar findings have been reported for PRRSV, where viral antigen and RNA were detected in both macrophages and granulosa cell layers [35]. The detection of inhibin‐α in only a single animal may reflect the known cyclic expression in granulosa cells [36]. Infection of granulosa cells could have functional implications for follicular development, luteal maintenance, and hormonal regulation, potentially contributing to reproductive failure independently of fetal infection [37].

The restriction of viral antigen to the fetal compartment at the feto‐maternal interface suggests that, at the time of examination, viral replication was largely confined to fetal tissues, while the maternal side was either not infected or had already cleared the virus. This observation may indicate that the porcine placental barrier limits viral spread towards maternal tissues at later stages of infection [38]. In contrast, the clinical course indicates that reproductive failure was closely associated with systemic maternal diseases. In most gilts, the onset of fever coincided with abortion or embryonic resorption, suggesting that maternal disease progression rather than local placental infection alone is a key driver of pregnancy loss [39].

Taken together, the present findings provide pathological evidence supporting the possibility of vertical transmission of ASFV following AI and highlight a complex interplay between maternal infection dynamics, fetal susceptibility, and viral tropism.

This study has several limitations. The number of animals and evaluable conceptuses was limited, and advanced autolysis precluded the histopathological assessment of tissues in the conceptus. In addition, sampling was restricted to defined time points, limiting conclusions regarding the precise temporal sequence of maternal infection and fetal exposure. In particular, it cannot be determined whether the absence of detectable viral antigen reflects the true absence of infection or a prior infection followed by viral clearance at the feto‐maternal interface. Kinetic studies during the peri‐implantation period around day 12–28 [40] would be required to directly assess viral dynamics at the feto‐maternal interface. Furthermore, the identity of infected fetal target cells could not be conclusively determined using the applied marker panel. Future studies including multiple gestational stages may provide further insights into ASFV pathogenesis and facilitate identification of fetal target cell populations once cellular differentiation is more advanced [41].

## 8. Conclusions

This study provides pathological evidence consistent with maternal‐to‐conceptus transmission of ASFV following AI with contaminated semen. ASFV infection of conceptuses was widespread yet highly heterogeneous and could occur even in the absence of detectable maternal antigen at later time points, consistent with temporally dynamic infection kinetics. The detection of viral antigen and nucleic acids in multiple conceptus tissues indicates systemic infection, while the limited characterization of infected cells by classical macrophage markers suggests a broader and incompletely defined cellular tropism in the early development. In the female reproductive tract, the localization of viral antigen supports the concept that local viral replication may occur and may represent a source of virus for infection of the conceptus, particularly at the early stages of pregnancy. Together, these findings highlight the complexity of ASFV transmission and pathogenesis during early pregnancy and suggest that multiple routes of maternal‐to‐conceptus transmission may be involved. Further studies are required to resolve infection dynamics at the feto‐maternal interface and to identify fetal target cell populations.

## Funding

This research has received funding through the Horizon 2020 ERA‐NET Cofund International Coordination of Research on Infectious Animal Diseases (ICRAD), project “ASF‐RASH” (Grant 82), the FLI ASF Research Network, and in part by the National Pork Board (Grant 21‐134), a program sponsored by the United States Department of Agriculture.

## Ethics Statement

The study was performed in accordance with the current German Animal Welfare regulations and approved by the competent authority (Landesamt für Landwirtschaft, Lebensmittelsicherheit und Fischerei Mecklenburg‐Vorpommern [LALLF M‐V]) under Reference Number 7221.3‐1‐071/21.

## Conflicts of Interest

The authors declare no conflicts of interest.

## Supporting Information

Additional supporting information can be found online in the Supporting Information section.

## Supporting information


**Supporting Information** Table S1: Integrated summary of clinical, virological, and histopathological findings in gilts following artificial insemination with ASFV‐contaminated semen. Abbreviations:  ^∗^, euthanized due to severe clinical signs; #, histological detection only; +, positive; −, negative; /, not applicable or not available; d.p.ins., days postinsemination; r, antigen detected in reproductive organs; s, antigen detected in standard organs.

## Data Availability

The data that support the findings of this study are available from the corresponding author upon reasonable request.
