# Supplementary material for: Infection of the Conceptus With African Swine Fever Virus Following Artificial Insemination in Sows: A Pathological Study
Source: Transbound Emerg Dis. 2026 Jun 29;2026:7164197. doi: 10.1155/tbed/7164197 (PMC13315835; doi:10.1155/tbed/7164197)
Supplement: Supplementary file 1 — Supporting Information Table S1: Integrated summary of clinical, virological, and histopathological findings in gilts following artificial insemination with ASFV‐contaminated semen. Abbreviations: ∗, euthanized due to severe clinical signs; #, histological detection only; +, positive; −, negative; /, not applicable or not available; d.p.ins., days postinsemination; r, antigen detected in reproductive organs; s, antigen detected in standard organs. [file TBED-2026-7164197-s001.docx]

**Supplementary Table S1.** Integrated summary of clinical, virological, and histopathological findings in gilts following artificial insemination with ASFV-contaminated semen. *Legend: *= euthanized due to severe clinical signs; d.p.ins.= days post insemination; #= histological detection only; s= antigen detected in standard organs; r= antigen detected in reproductive organs; + = positive; – = negative; / = not applicable or not available*.

| #gilt | necropsy (d.p.ins.) | infection kinetics | repro-ductive outcome | conceptus/ residues | maternal antigen (s = standard organs / r = reproductive organs) | antigen detection in conceptus (+/-) |
| --- | --- | --- | --- | --- | --- | --- |
| 539 | 9 | 1^st^ insemination | pregnant | absent | s / r | / |
| 567 | 9 | 1^st^ insemination | pregnant | absent | s / r | / |
| 536 | *17 | 1^st^ insemination | pregnant | #present | - | - |
| 534 | *20 | 1^st^ insemination | pregnant | #present | - | + |
| 627 | *33 | 2nd insemination or contact | pregnant | abortion (sampled) | s / r | - |
| 530 | 35 | 1^st^ insemination | not pregnant | - | s / r | - |
| 533 | 35 | 2nd insemination or contact | pregnant | present | - | + |
| 614 | 35 | 2nd insemination or contact | pregnant | absent | - | / |
| 576 | 35 | 2nd insemination or contact | pregnant | absent | - | / |
| 646 | 35 | 2nd insemination or contact | pregnant | absent | - | / |
| 635 | 36 | 2nd insemination or contact | pregnant | absent | - | / |
| 630 | 36 | 2nd insemination or contact | pregnant | abortion / no conceptus available | - | / |
| 610 | 36 | 1^st^ insemination | pregnant | absent | - | / |
| 654 | 36 | 2nd insemination or contact | pregnant | abortion / no conceptus available | - | / |
